# Assessing disability weights based on the responses of 30,660 people from four European countries

**DOI:** 10.1186/s12963-015-0042-4

**Published:** 2015-04-03

**Authors:** Juanita A Haagsma, Charline Maertens de Noordhout, Suzanne Polinder, Theo Vos, Arie H Havelaar, Alessandro Cassini, Brecht Devleesschauwer, Mirjam E Kretzschmar, Niko Speybroeck, Joshua A Salomon

**Affiliations:** Department of Public Health, Erasmus MC, P.O. Box 2040, , 3000, CA Rotterdam, The Netherlands; Institute for Health Metrics and Evaluation, University of Washington, Seattle, USA; Institute of Health and Society (IRSS), Université catholique de Louvain, Leuven, Belgium; National Institute for Public Health and the Environment, Centre for Infectious Disease Control, Bilthoven, the Netherlands; Emerging Pathogens Institute, University of Florida, Gainesville, Florida USA; Institute for Risk Assessment Sciences, Utrecht University, Utrecht, the Netherlands; European Centre for Disease Prevention and Control, Stockholm, Sweden; Department of Virology, Parasitology and Immunology, Faculty of Veterinary Medicine, Ghent University, Merelbeke, Belgium; Julius Center for Health Sciences and Primary Care, University Medical Center Utrecht, Utrecht, The Netherlands; Department of Global Health and Population, Harvard School of Public Health, Boston, USA

**Keywords:** Value of life, Disability weight, Disease burden, Disability adjusted life years, Summary measure of population health, Prioritisation

## Abstract

**Background:**

In calculations of burden of disease using disability-adjusted life years, disability weights are needed to quantify health losses relating to non-fatal outcomes, expressed as years lived with disability. In 2012 a new set of global disability weights was published for the Global Burden of Disease 2010 (GBD 2010) study. That study suggested that comparative assessments of different health outcomes are broadly similar across settings, but the significance of this conclusion has been debated. The aim of the present study was to estimate disability weights for Europe for a set of 255 health states, including 43 new health states, by replicating the GBD 2010 Disability Weights Measurement study among representative population samples from four European countries.

**Methods:**

For the assessment of disability weights for Europe we applied the GBD 2010 disability weights measurement approach in web-based sample surveys in Hungary, Italy, Netherlands, and Sweden. The survey included paired comparisons (PC) and population health equivalence questions (PHE) formulated as discrete choices. Probit regression analysis was used to estimate cardinal values from PC responses. To locate results onto the 0-to-1 disability weight scale, we assessed the feasibility of using the GBD 2010 scaling approach based on PHE questions, as well as an alternative approach using non-parametric regression.

**Results:**

In total, 30,660 respondents participated in the survey. Comparison of the probit regression results from the PC responses for each country indicated high linear correlations between countries. The PHE data had high levels of measurement error in these general population samples, which compromises the ability to infer ratio-scaled values from discrete choice responses. Using the non-parametric regression approach as an alternative rescaling procedure, the set of disability weights were bounded by distance vision mild impairment and anemia with the lowest weight (0.004) and severe multiple sclerosis with the highest weight (0.677).

**Conclusions:**

PC assessments of health outcomes in this study resulted in estimates that were highly correlated across four European countries. Assessment of the feasibility of rescaling based on a discrete choice formulation of the PHE question indicated that this approach may not be suitable for use in a web-based survey of the general population.

**Electronic supplementary material:**

The online version of this article (doi:10.1186/s12963-015-0042-4) contains supplementary material, which is available to authorized users.

## Background

Priority-setting for health care policies and research is informed increasingly by burden of disease and injury studies, because these studies provide knowledge on the size of health problems and the potential benefit of proposed interventions and policies directed against these problems [1,2]. Burden of disease can be expressed in disability-adjusted life years (DALYs), a summary measure of population health that captures health losses associated with mortality and with different non-fatal outcomes of diseases and injuries in a single figure [3-5]. The DALY methodology was developed in the 1990s for the Global Burden of Disease (GBD) study [6-10] and has since been used in many other disease burden studies (e.g. [11-16]) as well as in cost-utility studies (e.g.[17-19]).

DALYs are calculated by adding years of life lost (YLLs) and years lived with disability (YLDs). YLLs represent the life years lost due to premature death and are calculated for any cause by multiplying the number of deaths by a standardized expectation of remaining life years at the age of death. YLDs represent the life years lost due to disability, adjusted for the severity of the disability. YLDs are computed for a given health outcome by multiplying the prevalence of that outcome by a disability weight that has a value between 0 (equivalent to full health) and 1 (equivalent to death).

For the 1996 revision of the GBD a large set of global disability weights was derived in a group exercise in which a panel of health experts assessed conditions using a range of techniques, and the scale was determined largely by responses to two different variants of a measurement method called the person trade-off [3,20]. This approach has been criticized, particularly regarding aspects such as the health construct, measurement techniques, and panel composition [21-23]. Because of a need to improve the approach and a need for disability weights that reflect the views of the global population, a new approach to measuring disability weights was developed for the GBD 2010 study [24,25]. This study used a conceptually less difficult measurement technique to elicit health state valuations (paired comparisons instead of the person trade-off). Health state descriptions focused primarily on the impact of a condition on functional health status. The study collected responses from 30,230 people in 167 countries. For five countries (Bangladesh, Indonesia, Peru, Tanzania, and the United States of America) household sample surveys were used, with samples designed to be representative of the population in a particular geographical area (or in the case of the USA, nationally representative). An important finding of the GBD 2010 disability weights study was that comparative assessments of different disabling sequelae, as revealed in paired comparisons, are similar in samples that vary with respect to cultural, educational, environmental, and demographic circumstances [25]. The GBD 2010 disability weights study has been criticized regarding the estimated weights for certain conditions, such as vision loss, and for the interpretation of evidence on the level of international agreement in paired comparison responses [26,27].

For some purposes in which the need for standardization and global comparison is not primary, it is useful to have disability weights that reflect the particular views of a specific population under study, for example in a national burden of disease study [28]. The present study was initiated as part of a study on the burden of communicable diseases in the European Union/European Economic Area (EEA)/European Free Trade Association (EFTA) countries [29,30], which motivates an interest in disability weights from European population samples. The GBD 2010 disability weights study did include respondents from European countries; however, these respondents were not representative for these European countries, as they participated in an open access web-based survey rather than in nationally representative sample surveys. This raises a question as to whether the current GBD 2010 disability weights are suitable for national burden of disease studies in European countries.

The objectives of the present study were to:

1. Assess the feasibility of replicating the GBD 2010 disability weights measurement study in a set of four nationally representative sample surveys in European countries using web-based surveys.
2. Estimate disability weights for Europe for a set of 255 health states, including 43 new health states.
3. Evaluate consistency in comparative assessments of disability across selected European countries.

## Materials and methods

### Study design

For the assessment of a set of disability weights for Europe we replicated the online survey protocol used in the GBD 2010 disability weights measurement study [25].

### Health states and description

In total 255 health states were evaluated. These health states can be subdivided into four categories: original GBD 2010 health states (n = 172) [25], new health states (n = 43), modified GBD 2010 health states (n = 33), and health states that were included for experimental purposes but were not part of the European disability weights study (n = 7).

Regarding the original GBD 2010 health states, we selected all health states associated with infectious diseases, injuries, and vision and hearing loss—of primary interest for the new European study on communicable disease—and supplemented these health states with a further subset of GBD 2010 health states selected to have some representation from each of the other health state categories (e.g., cancer, cardiovascular and circulatory disease, diabetes, digestive and genitourinary disease, chronic respiratory disease, musculoskeletal disorders, neurological disorders, and other).

For the 43 new health states lay descriptions were constructed following the same general design principles used in GBD 2010. The descriptions have a word limit of 70 words or less and were constructed through an iterative process. The brief lay descriptions are intended to highlight the major functional consequences and symptoms associated with the health state using simple, non-clinical vocabulary. Disease experts and health professionals were consulted to ensure that the descriptions were appropriate and reflective of the common manifestations of the disabling sequela in question.

For the 33 modified health states the description of the health states of original GBD health states were amended because they were found to be lacking in consistency or in content [25,26]. For instance, in the case of spinal cord injury, incontinence was added to the description. Both the original and modified health state descriptions were evaluated in this study in order to facilitate direct comparison. The health state descriptions that were evaluated in this study are included in Additional file 1.

### Health state valuation

To elicit health state valuations for the 255 health states, two valuation techniques were used: paired comparison (PC) and population health equivalence (PHE). All of the 255 health states were evaluated with the PC technique, and a subset of 28 states were evaluated with PHE questions. Paired (sometimes called “pairwise”) comparison is an ordinal measurement method. With this method, persons in two alternative health states are presented, and respondents have to decide whom they regard as being healthier. PHE questions ask for a retrospective assessment that compares two hypothetical health programs. The first health program prevented 1,000 people from getting an illness that causes rapid death; the second health program prevented 1,500, 2,000, 3,000, 5,000, or 10,000 (dependent on the bid that was selected randomly for each question) people from getting an illness that is not fatal but causes the lifelong health problems of one of the selected health states. The respondents are asked to choose which health program they think produced the greater overall population health benefit.

The 28 health states that were evaluated here were a subset of the 30 health states evaluated with the PHE in the GBD 2010 disability weights study.

### Panel participants

The panel consisted of members of the general public aged 18 to 65 years from four European countries, namely Hungary, Italy, the Netherlands, and Sweden. We selected these four countries because they are believed to be representative of four regions of Europe (Eastern, Southern, Central, and Northern Europe) with regards to age, sex, and educational level. We used existing large internet panels in the selected European countries. By selecting panel members with certain characteristics (in our case: age, sex, and educational level) from the existing large panels, the panel of participants for this study could be composed in such a way that the respondents were representative of the population aged 18 to 65 in the selected countries. The procedure to invite panelists to fill in the questionnaire differed between the Netherlands and the other three countries. In the Netherlands panelists were invited via individual emails. In the three other countries a link to the questionnaire was placed on a website. Subsequently, the relevant respondents were selected based on their characteristics as assessed in the questionnaire. Because of this, the specific number of panelists that were invited to fill in the questionnaire in Hungary, Italy, and Sweden is not known, and the response rate could not be calculated for these countries.

### Data collection

The GBD 2010 disability weights study consisted of two main components: a) a face-to-face or telephone survey based on a subset of the sequelae (household survey) and b) a web-based survey based on the full set of sequelae. In the current study we used the GBD 2010 web-based survey instrument.

Three versions of the web-based survey were developed. The number and framing of the PC questions differed per version. Each version included questions regarding the demographics of the respondent (age, sex, educational and income level, and disease experience) and three PHE and PC questions. The first version of the questionnaire included 15 PC questions with a chronic framing, the second version included 15 PC questions with a temporary framing, and the third version included five PC questions with a chronic framing to accommodate PHE questions. Chronic framing means that the participants are asked to consider the situation that the described health state will last for the rest of a person’s life. Temporary framing means that the participant is asked to consider that the health state will last for one week.

The survey and description of health states were translated from English into Dutch, Hungarian, Italian, and Swedish using translation software and subsequently translated back into English. The translations were verified independently by bilingual native speakers.

In the period 23 September to 11 November 2013 the disability weight survey was administered via the internet. The survey versions and health states were randomly assigned to the respondents following a randomization algorithm. First, the algorithm randomly allocated the survey version, based on the lowest percentage of respondents at that moment for each version. After the version was allocated, the algorithm selected the health states based on the minimum number of allocations that the health state had at that moment, i.e., the probability of selection was inversely proportional to number of allocations that health state at that moment.

### Data analysis

Analyses were performed with R (version 3.0.2) [31] and SPSS (version 21). The PC data were analyzed through probit regression, following the approach used in GBD 2010 [25]. Coefficients from the probit regression were compared across the four European countries in order to assess variation in the comparative assessments of different disabilities, as expressed in paired comparisons. To examine the feasibility of using the PHE rescaling method from the GBD 2010, we evaluated the PHE data in terms of the probabilities of choosing the alternative program over the first program by health state and by bid, as well as by educational level. This analysis thus focused on “sensitivity to scope” in the PHE [32], i.e., the degree to which bid probabilities are dependent on the number of people benefiting from the program, as the conceptual model for analyzing PHE data presumes, as well as responsiveness to variation in the severity of the different outcomes under consideration, i.e., the degree to which bid probabilities are sensitive to the nature of the health outcomes affected by the two programs in each comparison. As an alternative rescaling procedure, we ran a non-parametric regression model (loess) of the probit regression coefficients against the logit-transformed disability weights from GBD 2010. Based on this loess fit, we then predicted logit transformed disability weights for each of the probit coefficients, including the ones that were not matched to a GBD 2010 health state. Finally, we applied an inverse logit transformation at the draw level to these predicted disability weights. Uncertainty intervals around the mean disability weights were estimated through a Monte Carlo simulation approach. First, 200 samples of the paired comparison coefficients were generated based on their probit estimated mean and standard deviation. These samples were then used to produce 200 loess fits, as described above. Based on each loess fit, 200 samples were generated for each of the disability weights, yielding a total of 40,000 samples per disability weight. Uncertainty intervals around the mean disability weights were derived as the 2.5th and 97.5th percentile of the corresponding distribution of sampled weights.

## Results

### Respondents

A total of 30,660 respondents filled in the questionnaire. Approximately half of the respondents were male. The average age was 42.3 (SD 13.1). 76% of the respondents had a low or medium educational level and the majority (84.9%) had a low to medium income level. Table 1 shows the characteristics of the respondents. The response rate in the Netherlands was 63.1%. The response rates of the other countries could not be calculated.

**Table 1:** **Characteristics of the 30,600 participants**

Sex	
Male	48.0%
Age (years)	
18-34	31.2%
35-49	35.1%
50-65	33.7%
Educational level	
Low	29.8%
Medium	45.7%
High	24.6%
Income level	
Low	39.5%
Medium	45.4%
High	15.1%
Country	
Hungary	19.8%
Italy	26.3%
Netherlands	26.2%
Sweden	27.8%

### Paired comparison

**Figure 1:**
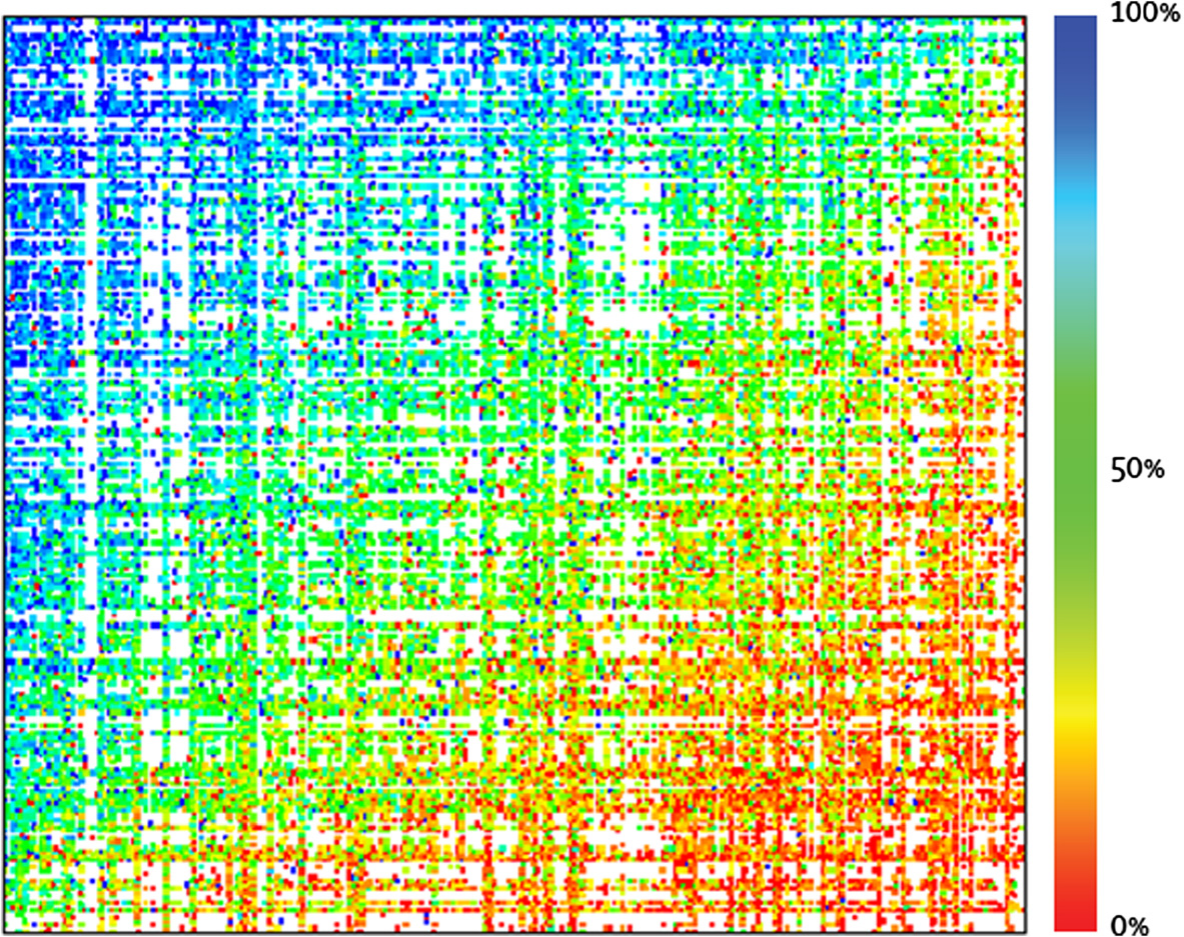
**Response probabilities for paired comparisons.** Red corresponds to probabilities that are 0.25 or lower. Blue corresponds to probabilities that are 0.75 or higher. Green, yellow, and orange correspond to probabilities between 0.25 and 0.75. A smooth transition in colors from high to low probabilities between the upper left and lower right corners indicates a small amount of measurement error and high internal consistency, whereas a completely random assortment of colors would indicate a high amount of measurement error and low internal consistency. It should be noted that not every possible 255 × 255 pair was evaluated with the pairwise comparison. This is indicated by the white spaces in the figure.

Figure 1 shows a heat map of the paired comparison response probabilities for the 255 × 255 possible paired comparisons. Each cell in the heat map indicates the response probability for one pair of states. The colors of the heat map correspond to the probability that the first health state in a pair comparison is chosen as the healthier outcome. Figure 1 shows a relatively smooth transition in colors from high to low probabilities between the upper left and lower right corner, indicating a small amount of measurement error and high internal consistency.

Of the respondents, 6.9% were given the same pair in the first and 15th paired comparison question, and of these 51% were presented in the same order and 49% in reversed order. This deliberate repetition allows assessment of test-retest reliability of PC responses. Overall, the probability of choosing the same health state was slightly higher if the two health states were presented in the same order (probability of choosing the same health state: 0.75) compared to reversed order (probability of choosing the same health state: 0.73). This is above the probability of chance agreement (0.50). The probabilities that respondents from Hungary (n = 414), Italy (n = 564), the Netherlands (n = 553), and Sweden (n = 573) chose the same health state in the retest were 0.78, 0.72, 0.73, and 0.75, respectively.

Comparison of the regression results on the paired comparison responses for each country with those run on the pooled data showed high linear correlations in all four cases (Pearson’s correlation coefficients between 0.855 and 0.978; p < 0.001; see Table 2).

**Table 2:** **Pearson’s correlation coefficients for country-specific and pooled probit regression analyses of paired comparison responses**

	**Hungary**	**Italy**	**Sweden**	**Pooled**
Netherlands	0.867*	0.855*	0.894*	0.941*
Hungary	-	0.944*	0.929*	0.966*
Italy		-	0.935*	0.967*
Sweden			-	0.978*

*correlation is significant at the 0.01 level.

### Population health equivalence

With the PHE a choice has to be made between two hypothetical health programs. We found that the probability of choosing the second health program option was higher as the bid increased (i.e., when the number of beneficiaries was greater), as expected. However, the span of probabilities between the lowest bid value (with 1,500 beneficiaries) and the highest bid value (with 10,000 beneficiaries) was generally lower than expected and varied by educational level on the PHE responses. On average, the differences between the probabilities of choosing the second health program at the highest versus the lowest bid values were 0.12, 0.16, and 0.19 for the lower, middle, and higher educational level, respectively.

The responsiveness to variation in the severity of the different outcomes under consideration was also lower than expected. While the 28 health states could be ranked according to the probabilities of choosing the second program (which prevented a specified number of cases of each outcome), there was relatively little variation across the range of health outcomes with quite distinct profiles of severity.

**Figure 2:**
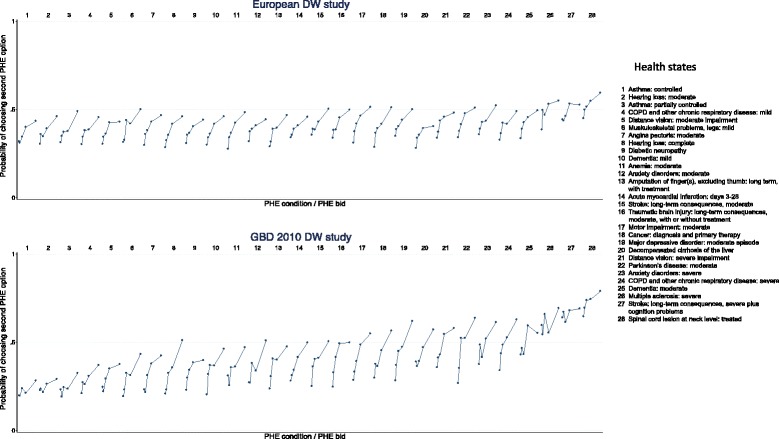
**Probability of choosing the second program at each bid value for each of the 28 health states that were evaluated with the population health equivalence questions, in the present study (top panel) compared to results in the GBD 2010 study (bottom panel).** Each line represents one health state and each dot represents a bid within one health state.

Figure 2 shows the probabilities of choosing the second program at each bid value for each of the 28 health states that were evaluated with the PHE. For comparison, a similar graph of the PHE data from the GBD 2010 disability weights measurement study is presented. The graphs show that the GBD 2010 PHE data had better discrimination by bid (higher sensitivity to scope), illustrated by longer lines between the bids within one health state, as well as a better discrimination by health state (better responsiveness to variation in the severity of the different outcomes), illustrated by a steeper gradient across health states, moving from left to right. These results suggest that the PHE responses in the present study were subject to high levels of measurement error; consequently, the feasibility of using discrete choice formulation in general population web-based sample surveys may be questioned.

### Disability weights

Given the evident lack of feasibility of the discrete choice PHE in this sample, a non-parametric regression approach was used as an alternative rescaling procedure to locate results onto the 0-to-1 disability weight scale. The R-squared from that regression was 0.801, based on 172 health states that were in both studies. The resulting disability weights and 95% uncertainty interval (UI) are shown in Table 3 (original GBD 2010 health states, new health states, and modified GBD 2010 health states). Distance vision mild impairment and mild anemia shared the lowest disability weight (0.004) and severe multiple sclerosis had the highest disability weight (0.677).

**Table 3:** **Estimated disability weights with uncertainty intervals (UI)**

		**Disability weight (+ UI)**		
**Category** ^**1**^		**Mean**	**2.5%**	**97.5%**
	**Infectious diseases**			
Original	Infectious disease, acute episode, mild	0.007	0.005	0.01
Original	Infectious disease, acute episode, moderate	0.051	0.039	0.06
Original	Infectious disease, acute episode, severe	0.125	0.104	0.152
Original	Infectious disease, post-acute consequences (fatigue, emotional lability, insomnia)	0.217	0.179	0.251
Original	Diarrhea, mild	0.073	0.061	0.092
Original	Diarrhea, moderate	0.149	0.12	0.182
Original	Diarrhea, severe	0.239	0.202	0.285
Original	Epididymo-orchitis	0.176	0.143	0.208
Original	HIV cases, symptomatic, pre-AIDS	0.351	0.299	0.394
Original	HIV/AIDS cases, receiving ARV treatment	0.108	0.089	0.132
Original	AIDS cases, not receiving ARV treatment	0.574	0.518	0.635
Original	Ear pain	0.015	0.011	0.019
Original	Tuberculosis, not HIV infected	0.308	0.264	0.353
Original	Tuberculosis, HIV infected	0.383	0.345	0.435
Original	Tuberculosis of vertebrae	0.287	0.245	0.332
New	Subacute sclerosing panencephalitis – phase 1	0.088	0.07	0.108
New	Thrombocytopenic purpura	0.167	0.134	0.201
New	Lymphogranuloma Venereum - local infection	0.070	0.057	0.087
New	Subacute sclerosing panencephalitis – phase 2	0.276	0.235	0.323
New	Subacute sclerosing panencephalitis – phase 3	0.543	0.481	0.606
	**Cancer**			
Original	Cancer, diagnosis and primary therapy	0.265	0.222	0.303
Original	Cancer, metastatic	0.358	0.317	0.417
Original	Stoma	0.125	0.104	0.155
Original	Terminal phase, with medication (for cancers, end-stage kidney/liver disease)	0.515	0.459	0.572
Original	Terminal phase, without medication (for cancers, end-stage kidney/liver disease)	0.588	0.524	0.65
	**Cardiovascular and circulatory disease**			
Original	Acute myocardial infarction, days 3-28	0.098	0.08	0.121
Original	Angina pectoris, moderate	0.103	0.089	0.128
Original	Cardiac conduction disorders and cardiac dysrhythmias	0.295	0.258	0.343
Original	Heart failure, mild	0.052	0.041	0.063
Original	Heart failure, moderate	0.070	0.057	0.085
Original	Heart failure, severe	0.173	0.14	0.205
Original	Stroke, long-term consequences, moderate	0.075	0.059	0.093
Original	Stroke, long-term consequences, severe plus cognition problems	0.580	0.519	0.639
	**Diabetes, digestive, and genitourinary disease**			
Original	Diabetic neuropathy	0.165	0.134	0.199
Original	Chronic kidney disease (stage IV)	0.108	0.09	0.132
Original	End-stage renal disease, on dialysis	0.487	0.432	0.544
Original	End-stage renal disease, with kidney transplant	0.030	0.023	0.037
Original	Decompensated cirrhosis of the liver	0.163	0.136	0.194
Original	Crohn's disease or ulcerative colitis	0.221	0.184	0.26
Original	Infertility, primary	0.008	0.005	0.01
Original	Infertility, secondary	0.007	0.005	0.01
New	Heart burn & reflux “GERD”	0.038	0.029	0.046
New	Constipation	0.075	0.061	0.092
New	Vaginal discharge	0.018	0.013	0.022
New	Dyspareunia	0.022	0.017	0.027
New	Irritable bowel syndrome	0.062	0.05	0.077
New	Stress incontinence	0.032	0.024	0.038
	**Chronic respiratory diseases**			
Original	Asthma, controlled	0.020	0.015	0.024
Original	Asthma, partially controlled	0.045	0.035	0.055
Original	COPD and other chronic respiratory problems, mild	0.025	0.019	0.031
Original	COPD and other chronic respiratory problems, moderate	0.284	0.242	0.329
Original	COPD and other chronic respiratory problems, severe	0.418	0.367	0.468
	**Mental, behavioural, and substance abuse disorder**			
New	Harmful alcohol use	0.106	0.087	0.132
New	Alcohol use disorder, very mild	0.154	0.123	0.187
Original	Alcohol use disorder, mild	0.209	0.175	0.247
Original	Alcohol use disorder, moderate	0.357	0.309	0.41
Original	Alcohol use disorder, severe	0.500	0.457	0.567
New	Cannabis dependence, mild	0.043	0.033	0.052
Original	Cannabis dependence	0.191	0.147	0.235
New	Amphetamine dependence, mild	0.088	0.072	0.11
Original	Amphetamine dependence	0.474	0.417	0.531
New	Cocaine dependence, mild	0.131	0.107	0.163
Original	Cocaine dependence	0.493	0.444	0.549
New	Opioid dependence, mild	0.365	0.314	0.417
Original	Heroin and other opioid dependence	0.624	0.553	0.707
Original	Anxiety disorders, mild	0.045	0.035	0.054
Original	Anxiety disorders, moderate	0.119	0.098	0.15
Original	Anxiety disorders, severe	0.422	0.372	0.475
Modified	Major depressive disorder, mild episode	0.129	0.102	0.154
Original	Major depressive disorder, moderate episode	0.294	0.248	0.341
Original	Major depressive disorder, severe episode	0.571	0.509	0.635
Modified	Intellectual disability, borderline	0.014	0.01	0.017
Modified	Intellectual disability, mild	0.053	0.041	0.065
Modified	Intellectual disability, moderate	0.123	0.097	0.152
Modified	Intellectual disability, severe	0.141	0.112	0.174
Modified	Intellectual disability, profound	0.213	0.177	0.255
New	Borderline personality disorder	0.193	0.16	0.228
New	Somatoform disorder	0.144	0.116	0.174
	**Hearing and vision loss**			
Modified	Hearing loss, mild	0.011	0.007	0.014
Modified	Hearing loss, moderate	0.037	0.028	0.045
Modified	Hearing loss, severe	0.152	0.125	0.187
Modified	Hearing loss, profound	0.235	0.197	0.274
Modified	Hearing loss, mild, with ringing	0.027	0.021	0.034
Modified	Hearing loss, moderate, with ringing	0.070	0.056	0.087
Modified	Hearing loss, severe, with ringing	0.274	0.231	0.318
Modified	Hearing loss, profound, with ringing	0.242	0.204	0.288
Modified	Hearing loss, complete, with ringing	0.313	0.268	0.361
Original	Unilateral hearing loss	0.008	0.005	0.012
Original	Near vision impairment	0.012	0.008	0.015
Original	Distance vision, mild impairment	0.004	0.002	0.005
Original	Distance vision, moderate impairment	0.034	0.027	0.042
Original	Distance vision, severe impairment	0.158	0.13	0.193
Modified	Distance vision blindness	0.173	0.145	0.213
	**Musculoskeletal disorders**			
Original	Back pain, acute, with leg pain	0.275	0.237	0.324
Original	Back pain, acute, without leg pain	0.298	0.254	0.343
Original	Back pain, chronic, with leg pain	0.395	0.345	0.45
Original	Back pain, chronic, without leg pain	0.365	0.322	0.413
New	Low back pain, mild	0.024	0.018	0.03
New	Low back pain, moderate	0.060	0.05	0.074
Original	Neck pain, acute, mild	0.062	0.05	0.075
Original	Neck pain, acute, severe	0.224	0.19	0.268
Original	Neck pain, chronic, mild	0.111	0.089	0.136
New	Neck pain, moderate	0.056	0.044	0.067
Original	Neck pain, chronic, severe	0.311	0.263	0.359
Original	Musculoskeletal problems, lower limbs, mild	0.027	0.021	0.032
Original	Musculoskeletal problems, lower limbs, moderate	0.094	0.08	0.12
Original	Musculoskeletal problems, lower limbs, severe	0.134	0.11	0.165
Original	Musculoskeletal problems, upper limbs, mild	0.041	0.032	0.05
Original	Musculoskeletal problems, upper limbs, moderate	0.138	0.114	0.167
Original	Musculoskeletal problems, generalized, moderate	0.344	0.3	0.391
Original	Musculoskeletal problems, generalized, severe	0.518	0.457	0.576
New	Osteomyelitis	0.053	0.041	0.065
New	Shoulder lesions	0.016	0.012	0.02
	**Injuries**			
Modified	Amputation of finger(s), excluding thumb	0.007	0.005	0.009
Original	Amputation of thumb (long term)	0.015	0.011	0.018
New	Amputation of one upper limb (long term, without treatment)	0.105	0.085	0.128
Modified	Amputation of one upper limb (with treatment)	0.048	0.037	0.057
Modified	Amputation of both upper limbs (long term, with treatment)	0.121	0.097	0.153
Modified	Amputation of both upper limbs (long term, without treatment)	0.392	0.344	0.451
Modified	Amputation of one lower limb (long term, with treatment)	0.041	0.031	0.049
Original	Amputation of one lower limb (long term, without treatment)	0.188	0.153	0.225
Modified	Amputation of both lower limbs (long term, with treatment)	0.088	0.071	0.107
Modified	Amputation of both lower limbs (long term, without treatment)	0.427	0.381	0.484
Original	Amputation of toe	0.007	0.005	0.009
Original	Burns, <20% total burned surface area without lower airway burns (short term, with or without treatment)	0.154	0.125	0.189
Original	Burns, <20% total burned surface area or <10% total burned surface area if head/neck or hands/wrist involved (long term, with or without treatment)	0.019	0.014	0.024
Original	Burns, ≥20% total burned surface area (short term, with or without treatment)	0.262	0.218	0.303
Original	Burns, ≥20% total burned surface area or ≥10% total burned surface area if head/neck or hands/wrist involved (long term, with treatment)	0.161	0.131	0.195
Original	Burns, ≥20% total burned surface area or ≥10% total burned surface area if head/neck or hands/wrist involved (long term, without treatment)	0.424	0.372	0.478
Original	Crush injury (short or long term, with or without treatment)	0.138	0.112	0.169
Original	Dislocation of hip (long term, with or without treatment)	0.018	0.014	0.023
Original	Dislocation of knee (long term, with or without treatment)	0.112	0.094	0.141
Original	Dislocation of shoulder (long term, with or without treatment)	0.041	0.033	0.051
Original	Other injuries of muscle and tendon (includes sprains, strains and dislocations other than shoulder, knee, hip)	0.009	0.007	0.012
Original	Drowning and nonfatal submersion (short or long term, with or without treatment)	0.240	0.197	0.286
Original	Fracture of clavicle, scapula or humerus (short or long term, with or without treatment)	0.038	0.029	0.045
Modified	Fracture of face bone (short or long term with or without treatment)	0.038	0.031	0.044
Original	Fracture of foot bones (short term, with or without treatment)	0.027	0.021	0.033
Original	Fracture of foot bones (long term, without treatment)	0.026	0.019	0.032
Original	Fracture of hand (short term, with or without treatment)	0.010	0.007	0.013
Original	Fracture of hand (long term, without treatment)	0.020	0.015	0.026
Original	Fracture of neck of femur (short term, with or without treatment)	0.228	0.193	0.275
Original	Fracture of neck of femur (long term, with treatment)	0.057	0.045	0.068
Original	Fracture of neck of femur (long term, without treatment)	0.440	0.391	0.493
Original	Fracture of patella, tibia or fibula or ankle (short term, with or without treatment)	0.044	0.034	0.053
Original	Fracture of patella, tibia or fibula or ankle (long term, with or without treatment)	0.051	0.04	0.062
Original	Fracture of pelvis (short term)	0.205	0.171	0.243
Original	Fracture of pelvis (long term)	0.158	0.127	0.194
Original	Fracture of radius or ulna (short term, with or without treatment)	0.030	0.024	0.037
Original	Fracture of radius or ulna (long term, without treatment)	0.052	0.042	0.063
Original	Fracture of skull (short or long term, with or without treatment)	0.083	0.066	0.103
Original	Fracture of sternum and/or fracture of one or two ribs (short term, with or without treatment)	0.185	0.161	0.21
Original	Fracture of vertebral column (short or long term, with or without treatment)	0.101	0.084	0.124
Original	Fracture, other than femoral neck (short term, with or without treatment)	0.080	0.064	0.097
Original	Fracture, other than femoral neck (long term, without treatment)	0.042	0.032	0.051
Original	Fractures, treated (long term)	0.005	0.004	0.008
Original	Injured nerves (short term)	0.126	0.104	0.156
Original	Injured nerves (long term)	0.074	0.059	0.088
Original	Injury to eyes (short term)	0.060	0.048	0.072
New	Concussion	0.104	0.085	0.126
Original	Traumatic brain injury, long-term consequences, minor (with or without treatment)	0.089	0.072	0.109
Original	Traumatic brain injury, long-term consequences, moderate (with or without treatment)	0.214	0.18	0.252
Original	Severe traumatic brain injury, short term (with or without treatment)	0.192	0.151	0.228
Original	Traumatic brain injury, long-term consequences, severe (with or without treatment)	0.604	0.539	0.674
Original	Open wound (short term, with or without treatment)	0.007	0.005	0.01
Original	Poisoning (short term with or without treatment)	0.170	0.139	0.202
Original	Severe chest injury (short term, with or without treatment)	0.377	0.333	0.434
Original	Severe chest injury (long term, with or without treatment)	0.047	0.036	0.056
Modified	Spinal cord lesion below neck level (treated)	0.298	0.256	0.349
Modified	Spinal cord lesion below neck level (untreated)	0.619	0.553	0.696
Modified	Spinal cord lesion at neck level (treated)	0.520	0.465	0.581
Modified	Spinal cord lesion at neck level (untreated)	0.648	0.578	0.728
	**Neurological disorders**			
Original	Dementia, mild	0.059	0.048	0.073
Original	Dementia, moderate	0.434	0.38	0.481
New	Encephalopathy - moderate	0.410	0.358	0.47
New	Encephalopathy - severe	0.447	0.391	0.501
New	Epilepsy, seizures > = once a month	0.488	0.432	0.546
New	Epilepsy, seizures 1–11 per year	0.255	0.215	0.294
Original	Epilepsy, severe	0.562	0.505	0.631
Original	Epilepsy, treated, with recent seizures	0.335	0.294	0.388
Original	Multiple sclerosis, mild	0.160	0.128	0.195
Original	Multiple sclerosis, moderate	0.469	0.417	0.531
Original	Multiple sclerosis, severe	0.677	0.594	0.757
Original	Parkinson's disease, mild	0.016	0.012	0.022
Original	Parkinson's disease, moderate	0.239	0.205	0.286
Original	Parkinson's disease, severe	0.530	0.477	0.59
New	Trigeminal neuralgia	0.068	0.056	0.084
New	Vertigo and balance disorder (Menière, labyrinthitis)	0.097	0.079	0.119
	**Other**			
Original	Abdominopelvic problem, mild	0.018	0.013	0.022
Original	Abdominopelvic problem, moderate	0.123	0.1	0.15
Original	Abdominopelvic problem, severe	0.310	0.262	0.355
New	Allergic rhinitis (hay fever)	0.006	0.004	0.009
New	Anal fissure/abscess/fistula	0.082	0.066	0.1
Original	Anemia, mild	0.004	0.003	0.006
Original	Anemia, moderate	0.045	0.035	0.054
Original	Anemia, severe	0.118	0.098	0.145
New	Carpal tunnel syndrome	0.039	0.031	0.047
Original	Conjunctivitis without corneal scar	0.015	0.011	0.019
Modifed	Generic uncomplicated disease: anxiety about diagnosis	0.021	0.015	0.026
Original	Generic uncomplicated disease: worry and daily medication	0.070	0.057	0.088
New	Haemorrhoids	0.109	0.085	0.133
New	Hyperthyroidism	0.144	0.115	0.176
New	Hypothyroidism	0.022	0.017	0.028
New	Insomnia	0.023	0.017	0.028
New	Intensive care unit admission	0.655	0.579	0.727
New	Invasive device/drain	0.163	0.131	0.198
Original	Motor impairment, mild	0.011	0.008	0.014
Original	Motor impairment, moderate	0.053	0.042	0.064
Original	Motor impairment, severe	0.421	0.377	0.477
Modified	Motor plus cognitive impairments, mild	0.044	0.035	0.053
Modified	Motor plus cognitive impairments, moderate	0.185	0.154	0.223
Modified	Motor plus cognitive impairments, severe	0.494	0.438	0.557
New	Sleep apnoea	0.036	0.027	0.044
New	Varicose veins	0.020	0.016	0.025

^1^Original = original GBD 2010 health states [25]; New = new health states; Modified = modified GBD 2010 health states.

The results show that the disability weights are ranked logically; lowest disability weights were attributed to mild health states, such as mild hearing impairment (disability weight 0.011) and mild acute infectious disease (disability weight 0.007), and highest disability weights were attributed to severe health states, such as the terminal phase of cancer or chronic kidney disease without medication (disability weight 0.588) and untreated spinal cord lesion below neck level (disability weight 0.648). This is illustrated by increasing disability weights by level of severity within specific types of diseases. For example, mild diarrhea (disability weight 0.073) is rated lower than moderate diarrhea (disability weight 0.149) and severe diarrhea (disability weight 0.239).

### Comparison to GBD disability weights

For 141 (82.0%) of the 172 health states that were included in the European and GBD studies, the point estimate of the European disability weight fell within the 95% UI of the GBD 2010 disability weights. For 17 (10.1%) health states the European disability weights were higher than the upper bound, and for 11 (6.5%) health states the European disability weights were lower than the lower bound of the 95% UI from the GBD 2010 study.

In absolute terms, differences between GBD and European disability weights ranged from −0.165 (HIV, cases, symptomatic, pre-AIDS; GBD 2010 disability weight = 0.186, European disability weight = 0.351) to 0.185 (fracture of pelvis, short term; GBD 2010 disability weight = 0.390, European disability weight = 0.205). The relative difference ranged from 0% to 61%, with the highest relative differences generally appearing in cases of low disability weights (asthma controlled GBD 2010 disability weight = 0.009, European disability weight = 0.020; fractures treated, long term GBD 2010 disability weight = 0.003, European disability weight = 0.005).

## Discussion

This study aimed to assess disability weights for 255 health states. The resulting disability weights were ranked logically; the lowest disability weights were attributed to mild health states and the highest disability weights to severe health states. Furthermore, the results pointed to a high level of overall agreement in paired comparison responses across four countries, as indicated by high linear correlations in country-specific results from probit regression analyses.

### Strengths of the current study

Thus far, the largest European disability weights study, published in 2003, included 232 respondents [33]. Apart from a lower number of health states, different valuation techniques and sample size, the study of Schwarzinger et al. utilized a different panel composition, namely health professionals rather than a population panel [33]. Since burden of disease studies are used primarily as a tool for decision-making on resource allocation at a population level, it has been recommended to incorporate the views of the general public to inform decision-making in a democratic society [25,28]. However, the majority of previously performed disability weight studies asked health professionals to value health states. Studies that included both medical experts and members of the general public showed significant differences between disability weights derived from these two groups [34-36].

### Web-based survey

A limitation of this study is that we used a web-based survey to collect the data. Internet users tend to be more highly educated and younger than the general EU population [37]. We have tried to mitigate these limitations by using existing large internet panels in the selected European countries. By selecting panel members with certain characteristics (in our case, age, sex, and educational level) from the existing large panel, the panel of participants for this study could be composed in such a way that it was representative of the population aged 18 to 65 years in the selected countries. Our panel did not include participants older than 65 years. For the age groups over age 65 it was too difficult to find enough participants. The GBD 2010 disability weights study did include respondents aged 65 and older (approximately 5% of the total sample).

### Population health equivalence

Based on responses to population health equivalence questions, as expected, the probability of choosing the second health program option was higher with increasing bid (i.e., a higher number people that are prevented from getting a certain illness). However, the differences between the choice probabilities with the highest (10,000 people prevented from getting a certain illness) and lowest bids (1,500 people prevented from getting that illness) were small. The relatively small difference is consistent with large numbers of respondents answering randomly, which will drive all aggregate-level response probabilities toward 50% and thus dilute differences across types of outcomes (either defined by different numbers of beneficiaries or different severity of the health state under consideration). The spans in response probabilities between the low and high bids were smallest among those with lower education. In the GBD study, the PHE was included in the web-based survey as well [25]. However, the educational level of the respondents of the GBD study was much higher (93% with a higher education) compared to our study (25% with a higher education), and respondents to the GBD survey were a self-selected group who were evidently interested enough in the content of the survey to participate voluntarily. This may have resulted in greater attention to the question and care in weighing the responses, both of which are likely to have improved the signal-to-noise ratio in the responses. We conclude from the results in the present study that the discrete choice formulation of the PHE may not be suitable for use in a general population survey administered by the internet.

### Disability weights

The ranking of certain conditions seems counterintuitive. For instance, the disability weight for profound intellectual disability is lower than the disability weight for back pain. A possible explanation for this may be that brief lay descriptions were used to describe the major functional consequences and symptoms associated with the health state and that the disease label, indicating the cause of the health state, was removed from the description. The latter was a deliberate choice, because the disease label may elicit bias for stigmatizing conditions [25]. However, previous studies showed that including certain disease information in health state descriptions yields different values [38]. A second explanation may be the framing of the paired comparison. In the pairwise comparison respondents are asked to judge the level of health of the health states, and this may lead to bias if respondents consider some health states as not being associated with “being ill” [26].

For future health state valuation studies that use a similar design and a similar panel composition it is important to consider different techniques to anchor estimates from paired comparisons onto the disability weight scale, such as the time trade-off or the standard gamble. However, each of these existing techniques to measure health state preferences suffers from limitations that hamper their application in a study design where a web-based survey is used to collect health state valuations from a panel that consists of members of the general public. Alternatively, the disability weights may be recalibrated post-hoc by health professionals. Health professionals are argued to have the ability to make careful comparative judgments. However, an argument against the use of a panel composed of health professionals is that the disability weights will not entirely reflect the views of the global population, as has been recommended.

### Agreement between European disability weights and GBD 2010 disability weights

Given the lack of feasibility of the discrete choice PHE in this sample, an alternative rescaling procedure was applied based on non-parametric regression. It is important to note that as a result, this study does not include new information on tradeoffs between nonfatal and fatal outcomes, which are central to the rescaling of results to a unique 0-to-1 disability weights scale. We therefore emphasize that comparison of disability weights between this study and GBD 2010 should be understood as reflecting variation in comparative evaluations of different functional outcomes (as manifest in responses to paired comparison questions) rather than a complete assessment of differences in the valuation of nonfatal versus fatal health outcomes.

### Cultural differences

Similar to the GBD 2010 disability weights measurement study, our study aspired to quantify health loss as opposed to welfare loss [25]. Previous studies have shown that there are clear cultural differences in the ways people perceive health problems and how such problems affect their lives [39-43]. This was endorsed by Üstün et al., who found significant differences in ranking of health states between 14 countries [36]. Furthermore, the findings from Jelsma et al. suggest that the effect of cultural differences on health state valuations may be stronger among lay people compared to health professionals [35]. However, in the largest disability weights study thus far, Salomon et al. found that comparative paired comparisons of different functional outcomes produced similar results in samples that varied with respect to cultural, educational, environmental, and demographic circumstances [25]. The current study also found a high degree of consistency between countries, though it should be noted that all of the countries in our study were high-income European settings, so we caution against over-generalization of the significance of the findings. Apart from cultural differences, other differences between high- and low-income settings may also influence how people weigh different health outcomes. For example, we might hypothesize that diseases and injuries rated as less severe by experts in a high-income country could be rated as more burdensome by people in low-income settings. Further research is needed to gain greater insight into the effects of cultural differences on disability weights, particularly in low-income settings.

## Conclusions

Limitations notwithstanding, this study provided an opportunity to expand the evidence base on disability weights derived from the GBD disability weights measurement study, since PC assessments of health outcomes in this study resulted in estimates that were highly correlated across four European countries. Furthermore, the European disability weights study provided the opportunity to expand the set of health outcomes that will be covered in the burden of communicable disease study in the European Union/EEA/EFTA countries and the next revision of the GBD.

## Additional file

Additional file 1:
**Three versions of the** **web-based** **survey were developed.** Each version included questions regarding the demographics of the respondent (age, sex, educational and income level, and disease experience), PHE and PC questions. Table A2 shows overview of the questions per version of the survey.
