# Supplementary material for: Assessing disability weights based on the responses of 30,660 people from four European countries
Source: Popul Health Metr. 2015 Apr 3;13:10. doi: 10.1186/s12963-015-0042-4 (PMC4715333; doi:10.1186/s12963-015-0042-4)
Supplement: Additional file 1: — Three versions of the web-based survey were developed. Each version included questions regarding the demographics of the respondent (age, sex, educational and income level, and disease experience), PHE and PC questions. Table A2 shows overview of the questions per version of the survey. [file 12963_2015_42_MOESM1_ESM.docx]

Additional file

Three versions of the web-based survey were developed. Each version included questions regarding the demographics of the respondent (age, sex, educational and income level, and disease experience), PHE and PC questions. Table A2 shows overview of the questions per version of the survey.

Table A1. Overview of three versions of the web-based survey

| **Version 1** | **Version 2 (A+B)** | **Version 3** |
| --- | --- | --- |
| Demographics module, Part I | Demographics module, Part I | Demographics module, Part I |
| 15 paired comparisons | 15 paired comparisons,  Version A: chronic  Version B: temporary  (chosen at random with pre-specified probability) | 5 paired comparisons |
| Demographics Part II | Demographics Part II | Demographics Part II |
| 3 population equivalence questions | 3 population equivalence questions | 3 population equivalence questions |

Table A2 shows the list of health states (in alphabetical order) and health state descriptions that were evaluated in the European disability weights study. Table A2 also indicates which health state description was included in which version of the survey.

Table A2. Health states and descriptions that were evaluated and version of the survey the health state was included

| **Category** | **Health state** | **Lay description** | Version 1 | Version 2A | Version 2B | Version 3 |
| --- | --- | --- | --- | --- | --- | --- |
| GBD-original | Abdominopelvic problem, mild | has some pain in the belly that causes nausea but does not interfere with daily activities. |  | * | * | * |
| GBD-original | Abdominopelvic problem, moderate | has pain in the belly and feels nauseous. The person has difficulties with daily activities. |  | * | * | * |
| GBD-original | Abdominopelvic problem, severe | has severe pain in the belly and feels nauseous. The person is anxious and unable to carry out daily activities. |  | * | * | * |
| GBD-original | Acute myocardial infarction, days 3-28 | gets short of breath after heavy physical activity, and tires easily, but has no problems when at rest. The person has to take medication every day and has some anxiety. |  | * | * | * |
| GBD-original | AIDS cases, not receiving ARV treatment | has severe weight loss, weakness, fatigue, cough and fever, and frequent infections, skin rashes and diarrhea. |  | * | * | * |
| GBD-original | Alcohol use disorder, mild | drinks a lot of alcohol and sometimes has difficulty controlling the urge to drink. While intoxicated, the person has difficulty performing daily activities. | * | * |  |  |
| GBD-original | Alcohol use disorder, moderate | drinks a lot, gets drunk almost every week and has great difficulty controlling the urge to drink. Drinking and recovering cause great difficulty in daily activities, sleep loss, and fatigue. | * | * |  |  |
| GBD-original | Alcohol use disorder, severe | gets drunk almost every day and is unable to control the urge to drink. Drinking and recovering replace most daily activities. The person has difficulty thinking, remembering and communicating, and feels constant pain and fatigue. | * | * |  |  |
| GBD-original | Amphetamine dependence | uses stimulants (drugs) and has difficulty controlling the habit. The person sometimes has depression, hallucinations and mood swings, and has difficulty in daily activities. |  |  |  | * |
| GBD-Modified | Amputation of both lower limbs (long term, with treatment) | has lost part of both legs, leaving pain and tingling in the stumps. The person has two artificial legs that make moving around possible, with extra effort. |  | * |  | * |
| GBD-original | Amputation of both lower limbs (long term, with treatment) | has lost part of both legs, leaving pain and tingling in the stumps. The person has two comfortable artificial legs, which allow for movement. | * | * |  | * |
| GBD-Modified | Amputation of both lower limbs (long term, without treatment) | has lost part of both legs, leaving pain, tingling, and frequent sores in the stumps. The person has great difficulty moving around, has episodes of depression and anxiety, and needs help from others to do many daily activities. |  | * |  | * |
| GBD-original | Amputation of both lower limbs (long term, without treatment) | has lost part of both legs, leaving pain, tingling, and frequent sores in the stumps. The person has great difficulty moving around and has episodes of depression, anxiety and flashbacks to the injury. | * | * |  | * |
| GBD-Modified | Amputation of both upper limbs (long term, with treatment) | has lost part of both arms, leaving pain and tingling in the stumps. The person has two artificial arms that make it possible to do daily activities, with a great deal of extra effort. |  | * |  | * |
| GBD-original | Amputation of both upper limbs (long term, with treatment) | has lost part of both arms, leaving pain and tingling in the stumps and flashbacks from the injury. The person has comfortable artificial arms and is mostly independent. | * | * |  | * |
| GBD-Modified | Amputation of both upper limbs (long term, without treatment) | has lost part of both arms, leaving pain and tingling in the stumps. The person needs a great deal of help from others to do even basic daily activities such as eating and using the toilet, and the person is very limited in other activities. |  | * |  | * |
| GBD-original | Amputation of both upper limbs (long term, without treatment) | has lost part of both arms, leaving pain and tingling in the stumps and flashbacks from the injury. The person needs help with basic daily activities such as eating and using the toilet. | * | * |  | * |
| GBD-Modified | Amputation of finger(s), excluding thumb | has lost a finger of one hand. At times there is pain and tingling in the stump. |  | * |  | * |
| GBD-original | Amputation of finger(s), excluding thumb (long term, with treatment) | has lost part of the fingers of one hand, causing difficulties in using the hand, pain, and tingling in the stumps. | * | * |  | * |
| GBD-Modified | Amputation of one lower limb (long term, with treatment) | has lost part of one leg, leaving pain and tingling in the stump. The person has an artificial leg that helps in moving around. |  | * |  | * |
| GBD-original | Amputation of one lower limb (long term, with treatment) | has lost part of one leg, leaving pain and tingling in the stump. The person has a comfortable artificial leg and only slight difficulties moving around. | * | * |  | * |
| GBD-original | Amputation of one lower limb (long term, without treatment) | has lost part of one leg, leaving pain and tingling in the stump. The person does not have an artificial leg, has frequent sores, and uses crutches. | * | * |  | * |
| GBD-original | Amputation of one upper limb (long term, with or without treatment) | has lost one hand and part of the arm, leaving pain and tingling in the stump and flashbacks from the injury. The person requires help lifting objects and in daily activities such as cooking. | * | * |  | * |
| GBD-Modified | Amputation of one upper limb (with treatment) | has lost one hand and part of the arm, leaving pain and tingling in the stump. The person has an artificial arm that makes it possible to lift objects and do daily activities such as cooking, with some extra effort. |  | * |  | * |
| GBD-original | Amputation of thumb (long term) | has lost one thumb, causing some difficulty in using the hand, pain, and tingling in the stump. | * | * |  | * |
| GBD-original | Amputation of toe | has lost one toe, leaving occasional pain and tingling in the stump. |  | * |  | * |
| GBD-original | Anemia, mild | feels slightly tired and weak at times, but this does not interfere with normal daily activities. | * | * | * | * |
| GBD-original | Anemia, moderate | feels moderate fatigue, weakness, and shortness of breath after exercise, making daily activities more difficult. | * | * | * | * |
| GBD-original | Anemia, severe | feels very weak, tired and short of breath, and has problems with activities that require physical effort or deep concentration. | * | * | * | * |
| GBD-original | Angina pectoris, moderate | has chest pain that occurs with moderate physical activity, such as walking uphill or more than half a kilometer (around a quarter-mile) on level ground. After a brief rest, the pain goes away. |  | * | * | * |
| GBD-original | Anxiety disorders, mild | feels mildly anxious and worried, which makes it slightly difficult to concentrate, remember things, and sleep. The person tires easily but is able to perform daily activities. | * | * | * |  |
| GBD-original | Anxiety disorders, moderate | feels anxious and worried, which makes it difficult to concentrate, remember things, and sleep. The person tires easily and finds it difficult to perform daily activities. | * | * | * |  |
| GBD-original | Anxiety disorders, severe | constantly feels very anxious and worried, which makes it difficult to concentrate, remember things and sleep. The person has lost pleasure in life and thinks about suicide. | * | * | * |  |
| GBD-original | Asthma, controlled | has wheezing and cough once a month, which does not cause difficulty with daily activities. |  | * |  | * |
| GBD-original | Asthma, partially controlled | has wheezing and cough once a week, which causes some difficulty with daily activities. |  | * |  | * |
| GBD-original | Back pain, acute, with leg pain | has severe back and leg pain, which causes difficulty dressing, sitting, standing, walking, and lifting things. The person sleeps poorly and feels worried. | * | * | * |  |
| GBD-original | Back pain, acute, without leg pain | has severe back pain, which causes difficulty dressing, sitting, standing, walking, and lifting things. The person sleeps poorly and feels worried. | * | * | * | * |
| GBD-original | Back pain, chronic, with leg pain | has constant back and leg pain, which causes difficulty dressing, sitting, standing, walking, and lifting things. The person sleeps poorly, is worried, and has lost some enjoyment in life. | * | * | * |  |
| GBD-original | Back pain, chronic, without leg pain | has constant back pain, which causes difficulty dressing, sitting, standing, walking, and lifting things. The person sleeps poorly, is worried, and has lost some enjoyment in life. | * | * | * | * |
| GBD-original | Borderline intellectual functioning | does not do well in school, has some difficulty doing complex or unfamiliar tasks, and has trouble concentrating. The person may also have behaviorial problems. | * | * |  | * |
| GBD-original | Burns, <20% total burned surface area or <*0% total burned surface area if head/neck or hands/wrist involved (long term, with or without treatment) | has scars caused by a burn. The scars are sometimes painful and itchy. | * | * |  | * |
| GBD-original | Burns, <20% total burned surface area without lower airway burns (short term, with or without treatment) | has a burn on part of the body. Parts of the burned area are painful, and other parts have lost feeling. |  |  | * |  |
| GBD-original | Burns, ≥20% total burned surface area (short term, with or without treatment) | has a painful burn over a large part of the body. Parts of the burned area have lost feeling, and the person feels anxious and unwell. |  |  | * |  |
| GBD-original | Burns, ≥20% total burned surface area or ≥*0% total burned surface area if head/neck or hands/wrist involved (long term, with treatment) | has scars caused by burns over a large part of the body. The scars are frequently painful and itchy, and the person is often sad. | * | * |  | * |
| GBD-original | Burns, ≥20% total burned surface area or ≥*0% total burned surface area if head/neck or hands/wrist involved (long term, without treatment) | has severe, disfiguring and itchy scars caused by burns over a large part of the body. The person cannot move some joints, feels sad, and has great difficulty with self-care such as dressing and toileting. | * | * |  | * |
| GBD-original | Cancer, diagnosis and primary therapy | has pain, nausea, fatigue, weight loss and high anxiety. |  | * | * | * |
| GBD-original | Cancer, metastatic | has severe pain, extreme fatigue, weight loss and high anxiety. |  | * | * | * |
| GBD-original | Cannabis dependence | uses marijuana daily and has difficulty controlling the habit. The person sometimes has mood swings, anxiety and hallucinations, and has some difficulty in daily activities. | * |  |  |  |
| GBD-original | Cardiac conduction disorders and cardiac dysrhythmias | has periods of rapid and irregular heartbeats and occasional fainting. |  | * | * | * |
| GBD-original | Chronic kidney disease (stage IV) | tires easily, has nausea, reduced appetite and difficulty sleeping. |  | * | * | * |
| GBD-original | Cocaine dependence | uses cocaine and has difficulty controlling the habit. The person sometimes has mood swings, anxiety, paranoia, hallucinations and sleep problems, and has some difficulty in daily activities. |  |  |  | * |
| GBD-original | Conjunctivitis without corneal scar | has eyes that are irritated, watery and sometimes itchy. |  |  | * |  |
| GBD-original | COPD and other chronic respiratory problems, mild | has cough and shortness of breath after heavy physical activity, but is able to walk long distances and climb stairs. |  | * | * | * |
| GBD-original | COPD and other chronic respiratory problems, moderate | has cough, wheezing and shortness of breath, even after light physical activity. The person feels tired and can walk only short distances or climb only a few stairs. |  | * | * | * |
| GBD-original | COPD and other chronic respiratory problems, severe | has cough, wheezing and shortness of breath all the time. The person has great difficulty walking even short distances or climbing any stairs, feels tired when at rest, and is anxious. |  | * | * | * |
| GBD-original | Crohn's disease or ulcerative colitis | has cramping abdominal pain, has diarrhea several times a day, and feels very tired for two months every year. When the person does not have symptoms, there is anxiety about them returning. | * | * |  | * |
| GBD-original | Crush injury (short or long term, with or without treatment) | had part of the body crushed, leaving pain, swelling, tingling and limited feeling in the affected area. |  | * |  | * |
| GBD-original | Decompensated cirrhosis of the liver | has a swollen belly and swollen legs. The person feels weakness, fatigue and loss of appetite. |  |  | * | * |
| GBD-original | Dementia, mild | has some trouble remembering recent events, and finds it hard to concentrate and make decisions and plans. | * | * |  | * |
| GBD-original | Dementia, moderate | has memory problems and confusion, feels disoriented, at times hears voices that are not real, and needs help with some daily activities. | * | * |  | * |
| GBD-original | Diabetic neuropathy | has pain, tingling and numbness in the arms, legs, hands and feet. The person sometimes gets cramps and muscle weakness. |  | * | * | * |
| GBD-original | Diarrhea, mild | has diarrhea three or more times a day with occasional discomfort in the belly. |  |  | * |  |
| GBD-original | Diarrhea, moderate | has diarrhea three or more times a day, with painful cramps in the belly and feeling thirsty |  |  | * |  |
| GBD-original | Diarrhea, severe | has diarrhea three or more times a day with severe belly cramps. The person is very thirsty and feels nauseous and tired. |  |  | * |  |
| GBD-original | Dislocation of hip (long term, with or without treatment) | walks with a limp and feels discomfort when walking. |  | * | * | * |
| GBD-original | Dislocation of knee (long term, with or without treatment) | has a knee out of joint, causing pain and difficulty moving the knee, which sometimes gives way. The person needs crutches for walking and help with self-care such as dressing. | * |  | * |  |
| GBD-original | Dislocation of shoulder (long term, with or without treatment) | has a shoulder that is out of joint, causing pain and difficulty moving. The person has difficulty with daily activities such as dressing and cooking. | * |  | * |  |
| GBD-Modified | Distance vision blindness | is completely blind, which causes great difficulty in some daily activities, and in going outside the home without assistance. The person sometimes feels worried or depressed due to social isolation. |  | * |  | * |
| GBD-original | Distance vision blindness | is completely blind, which causes great difficulty in some daily activities, worry and anxiety, and great difficulty going outside the home without assistance. | * |  |  |  |
| GBD-original | Distance vision, mild impairment | has some difficulty with distance vision, for example reading signs, but no other problems with eyesight. | * |  | * | * |
| GBD-original | Distance vision, moderate impairment | has vision problems that make it difficult to recognize faces or objects across a room. | * |  | * | * |
| GBD-original | Distance vision, severe impairment | has severe vision loss, which causes difficulty in daily activities, some emotional impact (for example worry), and some difficulty going outside the home without assistance. | * |  | * | * |
| GBD-original | Drowning and nonfatal submersion (short or long term, with or without treatment) | has breathlessness, anxiety, cough, and vomiting. |  | * | * |  |
| GBD-original | Ear pain | has an ear-ache that causes some difficulty with daily activities. | * |  | * | * |
| GBD-original | End-stage renal disease, on dialysis | is tired and has itching, cramps, headache, joint pains and shortness of breath. The person needs intensive medical care every other day lasting about half a day. |  | * | * | * |
| GBD-original | End-stage renal disease, with kidney transplant | sometimes feels tired and down, and has some difficulty with daily activities. |  | * | * | * |
| GBD-original | Epididymo-orchitis | has swelling and tenderness in the testicles and pain during urination. |  | * | * | * |
| GBD-original | Epilepsy, severe | has sudden, prolonged seizures once a week, with violent muscle contractions and stiffness, loss of consciousness, and loss of urine or stool control. Between seizures the person has drowsiness, memory loss, difficulty concentrating and anxiety |  | * |  | * |
| GBD-original | Epilepsy, treated, with recent seizures | has sudden seizures once a month, with violent muscle contractions and stiffness and loss of consciousness. Between seizures the person has some drowsiness, difficulty concentrating and anxiety about future episodes. |  | * |  | * |
| GBD-original | Fracture of clavicle, scapula or humerus (short or long term, with or without treatment) | has a broken shoulder bone, which is painful and swollen. The person cannot use the affected arm and has difficulty with getting dressed. |  |  | * |  |
| GBD-Modified | Fracture of face bone (short or long term with or without treatment) | has a broken cheek bone or a broken nose or chipped teeth, with swelling and severe pain. |  |  | * |  |
| GBD-original | Fracture of face bone (short or long term, with or without treatment) | has a broken cheek bone, broken nose, and chipped teeth, with swelling and severe pain. |  |  | * |  |
| GBD-original | Fracture of foot bones (long term, without treatment) | had a broken foot in the past that did not heal properly. The person now has pain in the foot and has some difficulty walking. |  | * | * | * |
| GBD-original | Fracture of foot bones (short term, with or without treatment) | has a broken foot bone, which causes pain, swelling, and difficulty walking. |  |  | * |  |
| GBD-original | Fracture of hand (long term, without treatment) | has stiffness in the hand and a weak grip. |  | * | * | * |
| GBD-original | Fracture of hand (short term, with or without treatment) | has a broken hand, causing pain and swelling. |  |  | * |  |
| GBD-original | Fracture of neck of femur (long term, with treatment) | had a broken hip in the past, which was fixed with treatment. The person can only walk short distances, has discomfort when moving around, and has some difficulty in daily activities. | * | * |  | * |
| GBD-original | Fracture of neck of femur (long term, without treatment) | had a broken hip bone in the past, which was never treated and did not heal properly. The person cannot get out of bed and needs help washing and going to the toilet. | * | * |  | * |
| GBD-original | Fracture of neck of femur (short term, with or without treatment) | has broken a hip and is in pain. The person cannot stand or walk, and needs help washing, dressing, and going to the toilet. |  |  | * |  |
| GBD-original | Fracture of patella, tibia or fibula or ankle (long term, with or without treatment) | had a broken shin bone in the past that did not heal properly. The person has pain in the knee and ankle, and has difficulty walking. | * | * |  | * |
| GBD-original | Fracture of patella, tibia or fibula or ankle (short term, with or without treatment) | has a broken shin bone, which causes severe pain, swelling, and difficulty walking. |  |  | * |  |
| GBD-original | Fracture of pelvis (long term) | had a broken pelvis in the past and now walks with a limp. There is often pain in the back and groin, and when urinating and sitting for a long time. | * | * |  | * |
| GBD-original | Fracture of pelvis (short term) | has a broken pelvis bone, with swelling and bruising. The person has severe pain, and cannot walk or do daily activities. |  |  | * |  |
| GBD-original | Fracture of radius or ulna (long term, without treatment) | had a broken forearm in the past that did not heal properly, causing some pain and limited movement in the elbow and wrist. The person has difficulty with daily activities such as dressing. | * | * |  | * |
| GBD-original | Fracture of radius or ulna (short term, with or without treatment) | has a broken forearm, which causes severe pain, swelling, and limited movement. |  |  | * |  |
| GBD-original | Fracture of skull (short or long term, with or without treatment) | has a broken skull, but does not have brain damage. The broken area is painful and swollen. |  |  | * |  |
| GBD-original | Fracture of sternum and/or fracture of one or two ribs (short term, with or without treatment) | has a broken rib that causes severe pain in the chest, especially when breathing in. The person has difficulty with daily activities such as dressing. |  |  | * |  |
| GBD-original | Fracture of vertebral column (short or long term, with or without treatment) | has broken back bones and is in pain, but still has full use of arms and legs. |  |  | * |  |
| GBD-original | Fracture, other than femoral neck (long term, without treatment) | had a broken thigh bone in the past, which was never treated and did not heal properly. The person now has a limp and discomfort when walking. | * | * |  | * |
| GBD-original | Fracture, other than femoral neck (short term, with or without treatment) | has a broken thigh bone. The person has severe pain and swelling and cannot walk. |  |  | * |  |
| GBD-original | Fractures, treated (long term) | has slight pain in a bone that was broken in the past. |  | * |  | * |
| GBD-Modified | Generic uncomplicated disease: anxiety about diagnosis | has a disease diagnosis that causes some worry but minimal interference with daily activities. |  | * |  | * |
| GBD-original | Generic uncomplicated disease: anxiety about diagnosis | has a disease diagnosis that causes worry about the future. |  | * |  | * |
| GBD-original | Generic uncomplicated disease: worry and daily medication | has a chronic disease that requires medication every day and causes some worry but minimal interference with daily activities. |  | * |  | * |
| GBD-Modified | Hearing loss, complete | cannot hear at all in any situation, including even the loudest sounds, and cannot communicate verbally or use a phone. Difficulties with communicating and relating to others often cause worry, depression or loneliness. |  | * |  | * |
| GBD-original | Hearing loss, complete | cannot hear at all, even loud sounds. | * | * |  | * |
| GBD-Modified | Hearing loss, complete, with ringing | cannot hear at all in any situation, including even the loudest sounds, and cannot communicate verbally or use a phone, and has very annoying ringing in the ears for more than half of the day. Difficulties with communicating and relating to others often cause worry, depression or loneliness. |  | * | * | * |
| GBD-original | Hearing loss, complete, with ringing | cannot hear at all, even loud sounds, cannot use a phone, and has ringing in the ears for more than 5 minutes, almost every day. |  | * | * | * |
| GBD-Modified | Hearing loss, mild | has great difficulty hearing and understanding another person talking in a noisy place (for example, on an urban street). |  | * |  | * |
| GBD-original | Hearing loss, mild | has difficulty following a conversation in a noisy environment but no other hearing problems. | * | * |  | * |
| GBD-Modified | Hearing loss, mild, with ringing | has great difficulty hearing and understanding another person talking in a noisy place (for example, on an urban street), and sometimes has annoying ringing in the ears. |  | * | * | * |
| GBD-original | Hearing loss, mild, with ringing | has great difficulty following a conversation in a noisy environment, and has ringing in the ears for more than 5 minutes, almost every day. |  | * | * | * |
| GBD-Modified | Hearing loss, moderate | is unable to hear and understand another person talking in a noisy place (for example, on an urban street), and has difficulty hearing another person talking even in a quiet place or on the phone. |  | * |  | * |
| GBD-original | Hearing loss, moderate | has difficulty hearing a normal voice and great difficulty following a conversation in a noisy environment. | * | * |  | * |
| GBD-Modified | Hearing loss, moderate, with ringing | is unable to hear and understand another person talking in a noisy place (for example, on an urban street), has difficulty hearing another person talking even in a quiet place or on the phone, and has annoying ringing in the ears for 5 minutes at a time, almost every day. |  | * | * | * |
| GBD-original | Hearing loss, moderate, with ringing | has difficulty hearing a normal voice or using a phone, has great difficulty following a conversation in a noisy environment, and has ringing in the ears for more than 5 minutes, almost every day. |  | * | * | * |
| GBD-Modified | Hearing loss, profound | is unable to hear and understand another person talking, even in a quiet place, is unable to take part in a phone conversation, and has great difficulty hearing anything in any other situation. Difficulties with communicating and relating to others often cause worry, depression or loneliness. |  | * |  |  |
| GBD-original | Hearing loss, profound | always has great difficulty hearing in any situation and is not able to use a phone. | * | * |  |  |
| GBD-Modified | Hearing loss, profound, with ringing | is unable to hear and understand another person talking, even in a quiet place, is unable to take part in a phone conversation, has great difficulty hearing anything in any other situation, and has annoying ringing in the ears for more than 5 minutes at a time, several times a day. Difficulties with communicating and relating to others often cause worry, depression, or loneliness. |  | * | * | * |
| GBD-original | Hearing loss, profound, with ringing | always has great difficulty hearing in any situation, cannot use a phone, and has ringing in the ears for more than 5 minutes, almost every day. |  | * | * | * |
| GBD-Modified | Hearing loss, severe | is unable to hear and understand another person talking, even in a quiet place, and unable to take part in a phone conversation. Difficulties with communicating and relating to others cause emotional impact at times (for example worry or depression). |  | * |  | * |
| GBD-original | Hearing loss, severe | has great difficulty hearing in any situation or in using a phone. |  | * |  | * |
| GBD-Modified | Hearing loss, severe, with ringing | is unable to hear and understand another person talking, even in a quiet place, is unable to take part in a phone conversation, and has annoying ringing in the ears for more than 5 minutes at a time, almost every day. Difficulties with communicating and relating to others cause emotional impact at times (for example worry or depression). |  | * | * | * |
| GBD-original | Hearing loss, severe, with ringing | has great difficulty hearing in any situation or in using a phone, and has ringing in the ears for more than 5 minutes, almost every day. |  | * | * | * |
| GBD-original | Heart failure, mild | is short of breath and easily tires with moderate physical activity, such as walking uphill or more than a quarter-mile on level ground. The person feels comfortable at rest or during activities requiring less effort. |  | * | * |  |
| GBD-original | Heart failure, moderate | is short of breath and easily tires with minimal physical activity, such as walking only a short distance. The person feels comfortable at rest but avoids moderate activity. |  | * | * |  |
| GBD-original | Heart failure, severe | is short of breath and feels tired when at rest. The person avoids any physical activity, for fear of worsening the breathing problems. | * | * | * |  |
| GBD-original | Heroin and other opioid dependence | uses heroin daily and has difficulty controlling the habit. When the effects wear off, the person feels severe nausea, agitation, vomiting and fever. The person has a lot of difficulty in daily activities. | * |  |  |  |
| GBD-original | HIV cases, symptomatic, pre-AIDS | has weight loss, fatigue, and frequent infections. |  | * | * | * |
| GBD-original | HIV/AIDS cases, receiving ARV treatment | has occasional fevers and infections. The person takes daily medication that sometimes causes diarrhea. |  | * | * | * |
| GBD-original | Infectious disease, acute episode, mild | has a low fever and mild discomfort , but no difficulty with daily activities. |  |  | * |  |
| GBD-original | Infectious disease, acute episode, moderate | has a fever and aches, and feels weak, which causes some difficulty with daily activities. |  |  | * |  |
| GBD-original | Infectious disease, acute episode, severe | has a high fever and pain, and feels very weak, which causes great difficulty with daily activities. |  |  | * |  |
| GBD-original | Infectious disease, post-acute consequences (fatigue, emotional lability, insomnia) | is always tired and easily upset. The person feels pain all over the body and is depressed. |  | * | * | * |
| GBD-original | Infertility, primary | wants to have a child and has a fertile partner, but the couple cannot conceive. | * | * |  | * |
| GBD-original | Infertility, secondary | has at least one child, and wants to have more children. The person has a fertile partner, but the couple cannot conceive. | * | * |  | * |
| GBD-original | Injured nerves (long term) | had a nerve injury in the past, which continues to cause some difficulty moving. The person often injures the affected part because it is numb. | * | * |  | * |
| GBD-original | Injured nerves (short term) | has a nerve injury, which causes difficulty moving and some loss of feeling in the affected area. |  |  | * |  |
| GBD-original | Injury to eyes (short term) | has an injury to one eye, which causes pain and difficulty seeing. |  |  | * |  |
| GBD-original | Intellectual disability / mental retardation, mild | has low intelligence and is slow in learning at school. As an adult, the person can work at simple supervised jobs and live independently, but often needs help to raise children. | * | * |  | * |
| GBD-original | Intellectual disability / mental retardation, moderate | has low intelligence and is slow in learning to speak and do simple tasks. As an adult, the person requires a lot of support to work productively, live independently and raise children. | * | * |  | * |
| GBD-original | Intellectual disability / mental retardation, profound | has low intelligence, cannot understand basic requests or instructions, and requires constant assistance for nearly all activities. | * | * |  | * |
| GBD-original | Intellectual disability / mental retardation, severe | has low intelligence and cannot speak more than a few words, needs help with most basic daily activities, and can do only simple tasks under close supervision. | * | * |  | * |
| GBD-Modified | Intellectual disability, borderline | is slow in learning at school. As an adult, the person has some difficulty doing complex or unfamiliar tasks but otherwise functions independently. |  | * |  | * |
| GBD-Modified | Intellectual disability, mild | has low intelligence and is slow in learning at school. As an adult, the person can live independently, but often needs help to raise children and can only work at simple supervised jobs. |  | * |  | * |
| GBD-Modified | Intellectual disability, moderate | has low intelligence, and is slow in learning to speak and to do even simple tasks. As an adult, the person requires a lot of support to live independently and raise children. The person can only work at the simplest supervised jobs. |  | * |  | * |
| GBD-Modified | Intellectual disability, profound | has very low intelligence, has almost no language, and does not understand even the most basic requests or instructions. The person requires constant supervision and help for all activities. |  | * |  | * |
| GBD-Modified | Intellectual disability, severe | has very low intelligence and cannot speak more than a few words, needs constant supervision and help with most daily activities, and can do only the simplest tasks. |  | * |  | * |
| GBD-Modified | Major depressive disorder, mild episode | feels persistent sadness and has lost interest in usual activities. The person sometimes sleeps badly, feels tired, or has trouble concentrating but still manages to function in daily life with extra effort. |  | * | * | * |
| GBD-original | Major depressive disorder, mild episode | has constant sadness and has lost interest in usual activities. The person can still function in daily life with extra effort, but sleeps badly, feels tired, and has trouble concentrating. | * | * | * | * |
| GBD-original | Major depressive disorder, moderate episode | has constant sadness and has lost interest in usual activities. The person has some difficulty in daily life, sleeps badly, has trouble concentrating, and sometimes thinks about harming himself (or herself). | * | * | * | * |
| GBD-original | Major depressive disorder, severe episode | has overwhelming, constant sadness and cannot function in daily life. The person sometimes loses touch with reality and wants to harm or kill himself (or herself). | * | * | * | * |
| GBD-original | Motor impairment, mild | has some difficulty in moving around but is able to walk without help. |  | * | * | * |
| GBD-original | Motor impairment, moderate | has some difficulty in moving around, and difficulty in lifting and holding objects, dressing and sitting upright, but is able to walk without help. |  | * | * | * |
| GBD-original | Motor impairment, severe | is unable to move around without help, and is not able to lift or hold objects, get dressed or sit upright. |  | * | * | * |
| GBD-Modified | Motor plus cognitive impairments, mild | has some difficulty in moving around but is able to walk without help. The person is slow in learning at school. As an adult, the person has some difficulty doing complex or unfamiliar tasks but otherwise functions independently. |  | * |  | * |
| GBD-original | Motor plus cognitive impairments, mild | has some difficulty in moving around, and is slow in learning at school. The person can walk without help, work at simple supervised jobs and live independently, but often needs help to raise children. |  | * |  | * |
| GBD-Modified | Motor plus cognitive impairments, moderate | has some difficulty in moving around, holding objects, dressing and sitting upright, but can walk without help. The person has low intelligence and is slow in learning to speak and to do simple tasks. As an adult, the person requires support to live independently and raise children and can only work at simple supervised jobs. |  | * |  | * |
| GBD-original | Motor plus cognitive impairments, moderate | has some difficulty in moving around, holding objects, dressing and sitting upright, and is slow in learning to speak and do simple tasks. The person can walk without help, but requires a lot of help with daily activities. |  | * |  | * |
| GBD-Modified | Motor plus cognitive impairments, severe | cannot move around without help, and cannot lift or hold objects, get dressed or sit upright. The person also has very low intelligence, speaks few words, and needs constant supervision and help with all daily activities. |  | * |  | * |
| GBD-original | Motor plus cognitive impairments, severe | cannot move around without help, and cannot lift or hold objects, get dressed or sit upright. The person also has low intelligence, speaks few words, and needs a lot of help with all basic daily activities. |  | * |  | * |
| GBD-original | Multiple sclerosis, mild | has mild loss of feeling in one hand, is a little unsteady while walking, has slight loss of vision in one eye, and often needs to urinate urgently. | * | * |  | * |
| GBD-original | Multiple sclerosis, moderate | needs help walking, has difficulty with writing and arm coordination, has loss of vision in one eye and cannot control urinating. | * | * |  | * |
| GBD-original | Multiple sclerosis, severe | has slurred speech and difficulty swallowing. The person has weak arms and hands, very limited and stiff leg movement, has loss of vision in both eyes and cannot control urinating. | * | * |  | * |
| GBD-original | Musculoskeletal problems, generalized, moderate | has pain and deformity in most joints, causing difficulty moving around, getting up and down, and using the hands for lifting and carrying. The person often feels fatigue. |  | * | * |  |
| GBD-original | Musculoskeletal problems, generalized, severe | has severe, constant pain and deformity in most joints, causing difficulty moving around, getting up and down, eating, dressing, lifting, carrying and using the hands. The person often feels sadness, anxiety and extreme fatigue. |  | * | * |  |
| GBD-original | Musculoskeletal problems, lower limbs, mild | has pain in the leg, which causes some difficulty running, walking long distances, and getting up and down. |  | * | * | * |
| GBD-original | Musculoskeletal problems, lower limbs, moderate | has moderate pain in the leg, which makes the person limp, and causes some difficulty walking, standing, lifting and carrying heavy things, getting up and down and sleeping. | * | * | * |  |
| GBD-original | Musculoskeletal problems, lower limbs, severe | has severe pain in the leg, which makes the person limp and causes a lot of difficulty walking, standing, lifting and carrying heavy things, getting up and down, and sleeping. | * | * | * |  |
| GBD-original | Musculoskeletal problems, upper limbs, mild | has mild pain and stiffness in the arms and hands. The person has some difficulty lifting, carrying and holding things. |  | * | * | * |
| GBD-original | Musculoskeletal problems, upper limbs, moderate | has moderate pain and stiffness in the arms and hands, which causes difficulty lifting, carrying, and holding things, and trouble sleeping because of the pain. | * | * | * |  |
| GBD-original | Near vision impairment | has difficulty seeing things that are nearer than 3 feet, but has no difficulty with seeing things at a distance. |  | * | * | * |
| GBD-original | Neck pain, acute, mild | has neck pain, and has difficulty turning the head and lifting things. |  | * |  | * |
| GBD-original | Neck pain, acute, severe | has severe neck pain, and difficulty turning the head and lifting things. The person gets headaches and arm pain, sleeps poorly, and feels tired and worried. | * |  | * |  |
| GBD-original | Neck pain, chronic, mild | has constant neck pain, and has difficulty turning the head, holding arms up, and lifting things |  | * |  | * |
| GBD-original | Neck pain, chronic, severe | has constant neck pain and arm pain, and difficulty turning the head, holding arms up, and lifting things. The person gets headaches, sleeps poorly, and feels tired and worried. | * |  | * |  |
| GBD-original | Open wound (short term, with or without treatment) | has a cut in the skin, which causes pain and numbness around the cut. |  |  | * |  |
| GBD-original | Other injuries of muscle and tendon (includes sprains, strains and dislocations other than shoulder, knee, hip) | has a strained muscle that causes pain and swelling. |  |  | * |  |
| GBD-original | Parkinson's disease, mild | has mild tremors and moves a little slowly, but is able to walk and do daily activities without assistance. | * | * |  | * |
| GBD-original | Parkinson's disease, moderate | has moderate tremors and moves slowly, which causes some difficulty in walking and daily activities. The person has some trouble swallowing, talking, sleeping, and remembering things. | * | * |  | * |
| GBD-original | Parkinson's disease, severe | has severe tremors and moves very slowly, which causes great difficulty in walking and daily activities. The person falls easily and has a lot of difficulty talking, swallowing, sleeping, and remembering things. | * | * |  | * |
| GBD-original | Poisoning (short term with or without treatment) | has drowsiness, stomach pain and vomiting. |  | * | * | * |
| GBD-original | Severe chest injury (long term, with or without treatment) | had a severe chest injury in the past that has now healed. The person still gets breathless when walking and feels discomfort in the chest. | * | * |  | * |
| GBD-original | Severe chest injury (short term, with or without treatment) | has a serious chest injury, which causes severe pain, shortness of breath and anxiety. |  |  | * |  |
| GBD-original | Severe traumatic brain injury, short term (with or without treatment) | cannot concentrate and has headaches, memory problems, dizziness, and feels angry. |  | * | * | * |
| GBD-Modified | Spinal cord lesion at neck level (treated) | is paralyzed from the neck down, with no feeling or control over any part of the body below the neck, and no urine or bowel control. |  | * |  | * |
| GBD-original | Spinal cord lesion at neck level (treated) | is paralyzed from the neck down and cannot feel or move the arms and legs. |  | * |  | * |
| GBD-Modified | Spinal cord lesion at neck level (untreated) | is paralyzed from the neck down, with no feeling or control over any part of the body below the neck, and no urine or bowel control. Arms and legs are in fixed, bent positions, and the person gets frequent infections and pressure sores. |  | * |  | * |
| GBD-original | Spinal cord lesion at neck level (untreated) | is paralyzed from the neck down and cannot feel or move the arms and legs. Arms and legs are in fixed, bent positions, and the person gets frequent infections and pressure sores. |  | * |  | * |
| GBD-Modified | Spinal cord lesion below neck level (treated) | is paralyzed from the waist down, cannot feel or move the legs and has difficulties with urine and bowel control. The person uses a wheelchair to move around. |  | * |  | * |
| GBD-original | Spinal cord lesion below neck level (treated) | is paralyzed from the waist down and cannot feel or move the legs. The person uses a lightweight and comfortable wheelchair to move around. |  | * |  | * |
| GBD-Modified | Spinal cord lesion below neck level (untreated) | is paralyzed from the waist down, cannot feel or move the legs and has difficulties with urine and bowel control. Legs are in fixed, bent positions, and the person gets frequent infections and pressure sores. |  | * |  | * |
| GBD-original | Spinal cord lesion below neck level (untreated) | is paralyzed from the waist down and cannot feel or move the legs. Legs are in fixed, bent positions, and the person gets frequent infections and pressure sores. |  | * |  | * |
| GBD-original | Stoma | has a pouch attached to an opening in the belly to collect and empty stools. |  | * |  | * |
| GBD-original | Stroke, long-term consequences, moderate | has some difficulty in moving around, and in using the hands for lifting and holding things, dressing and grooming. |  | * | * | * |
| GBD-original | Stroke, long-term consequences, severe plus cognition problems | is confined to bed or a wheelchair, depends on others for feeding, toileting and dressing, and has difficulty speaking, thinking clearly and remembering things. |  | * | * | * |
| GBD-original | Terminal phase, with medication (for cancers, end-stage kidney/liver disease) | has lost a lot of weight and regularly uses strong medication to avoid constant pain. The person has no appetite, feels nauseous, and needs to spend most of the day in bed. | * |  | * | * |
| GBD-original | Terminal phase, without medication (for cancers, end-stage kidney/liver disease) | has lost a lot of weight and has constant pain. The person has no appetite, feels nauseous, and needs to spend most of the day in bed. |  | * | * |  |
| GBD-original | Traumatic brain injury, long-term consequences, minor (with or without treatment) | has episodes of headaches, memory problems, and difficulty concentrating. | * |  | * | * |
| GBD-original | Traumatic brain injury, long-term consequences, moderate (with or without treatment) | has frequent headaches, memory problems, difficulty concentrating, and dizziness. The person is often anxious and moody. | * |  | * | * |
| GBD-original | Traumatic brain injury, long-term consequences, severe (with or without treatment) | cannot think clearly and has frequent headaches, memory problems, difficulty concentrating and dizziness. The person is often anxious and moody, and depends on others for feeding, toileting, dressing and walking. | * |  | * | * |
| GBD-original | Tuberculosis of vertebrae | has severe pain in the back and is unable to straighten the back. The person has lost weight and feels weak. |  | * | * |  |
| GBD-original | Tuberculosis, HIV infected | has a persistent cough and fever, shortness of breath, night sweats, weakness and fatigue and severe weight loss. |  | * | * | * |
| GBD-original | Tuberculosis, not HIV infected | has a persistent cough and fever, is short of breath, feels weak, and has lost a lot of weight. | * |  | * | * |
| GBD-original | Unilateral hearing loss | can hear well with one ear but has hearing loss in the other ear, resulting in some trouble following a conversation in a noisy environment. | * |  |  |  |
| New | Alcohol use disorder, very mild | drinks alcohol daily and has difficulty controlling the urge to drink. When sober, the person functions normally. |  | * |  |  |
| New | Allergic rhinitis (hay fever) | has an itchy, runny nose and frequent sneezing. |  |  | * |  |
| New | Amphetamine dependence, mild | uses stimulants (drugs) at least once a week and has some difficulty controlling the habit. When not using, the person functions normally. |  | * |  |  |
| New | Amputation of one upper limb (long term, without treatment) | has lost one hand and part of the arm, leaving pain and tingling in the stump. The person needs help from others to lift objects or do daily activities such as cooking. |  | * |  | * |
| New | Anal fissure/abcess/fistula | has pain around the anus that is worse when passing stools and sitting. |  |  | * |  |
| New | Borderline personality disorder | has unstable moods, often does things without thinking about the consequences and tends to view others and self as either very good or very bad. This leads to troubled relationships and causes the person to feel angry, anxious or depressed. |  | * | * | * |
| New | Cannabis dependence, mild | uses marijuana at least once a week and has some difficulty controlling the habit. When not using, the person functions normally. |  | * |  |  |
| New | Carpal tunnel syndrome | feels pain, numbness or weakness in part of the hand during some activities such as knitting, driving, writing or typing. |  | * | * | * |
| New | Cocaine dependence, mild | uses cocaine at least once a week and has some difficulty controlling the habit. When not using, the person functions normally. |  | * |  |  |
| New | Concussion | has headaches, dizziness, nausea and difficulty concentrating. |  |  | * |  |
| New | Constipation | passes stools infrequently and when it does happen it is painful. |  |  | * |  |
| New | Dyspareunia | experiences pain in the vagina during sexual intercourse. |  |  | * |  |
| New | Encephalopathy - moderate | has difficulty concentrating, confusion, fatigue and irritability. The person has difficulty with walking, speaking and personal care. |  | * | * | * |
| New | Encephalopathy - severe | has difficulty concentrating or speaking, confusion, fatigue and irritability. The person needs a great deal of help from others to do even basic daily activities such as eating and using the toilet, and the person is very limited in other activities. |  | * | * | * |
| New | Epilepsy, seizures >= once a month | has sudden seizures one or more times each month, with violent muscle contractions and stiffness, loss of consciousness, and loss of urine or bowel control. Between seizures the person has memory loss and difficulty concentrating. |  | * |  | * |
| New | Epilepsy, seizures *-** per year | has sudden seizures two to five times a year, with violent muscle contractions and stiffness, loss of consciousness, and loss of urine or bowel control. |  | * |  | * |
| New | Haemorrhoids | loses some blood when passing stools and at times has pain around the anus. |  |  | * |  |
| New | Harmful alcohol use | regularly gets drunk, to such an extent that the person may risk injury. |  |  | * |  |
| New | Heart burn & reflux “GERD” | often has a burning sensation in the back of the chest after eating. |  | * |  | * |
| New | Hyperthyroidism | feels nervous, has palpitations, sweats a lot and has difficulty sleeping. |  |  | * |  |
| New | Hypothyroidism | has low energy and feels cold. |  | * | * | * |
| New | Insomnia | has difficulty falling or staying asleep. |  | * | * | * |
| New | Intensive care unit admission | is very ill and often asleep or unconscious; when awake cannot move in bed, cannot speak, is completely dependent on others and is anxious. |  | * | * | * |
| New | Invasive device/drain | has a tube inserted in the body to drain fluid, which may cause skin irritation and difficulty in moving around. |  |  | * |  |
| New | Irritable bowel syndrome | has abdominal pain, bloating, flatulence and irregular bowel movements. |  |  | * |  |
| New | Low back pain, mild | has mild back pain, which causes some difficulty dressing, standing, and lifting things. |  | * | * | * |
| New | Low back pain, moderate | has moderate back pain, which causes difficulty dressing, sitting, standing, walking, and lifting things. |  | * | * | * |
| New | Lymphogranuloma Venereum *- local infection* | has a painful lump in the groin. |  |  | * |  |
| New | Neck pain, moderate | has moderate neck pain and difficulty turning the head and lifting things. The person gets headaches. |  | * | * | * |
| New | Opioid dependence, mild | uses heroin (or methadone) daily and has difficulty controlling the habit. When not using, the person functions normally. |  | * |  |  |
| New | Osteomyelitis | has severe pain in one leg that causes difficulty in moving about. |  | * | * | * |
| New | Shoulder lesions | has a painful shoulder that causes difficulty in using the arm. |  | * | * | * |
| New | Sleep apnoea | feels sleepy during the day and has difficulty concentrating. |  | * | * | * |
| New | Somatoform disorder | experiences pain, stomach problems or numbness and weakness in part of the body, and feels worry. |  |  | * |  |
| New | Stress incontinence | loses small amounts of urine without meaning to when coughing, sneezing, laughing or during physical exercise. |  | * | * | * |
| New | Subacute sclerosing panencephalitis – phase * | has difficulty concentrating, some memory problems, confusion and irritability. |  | * | * | * |
| New | Subacute sclerosing panencephalitis – phase 2 | has difficulty concentrating, some memory problems, confusion and irritability. The person has difficulty controlling body movements and moving around. |  | * | * | * |
| New | Subacute sclerosing panencephalitis – phase 3 | is often asleep or unconscious; when awake cannot think or see clearly. The person needs a great deal of help from others to do even basic daily activities such as eating and using the toilet, and the person is very limited in other activities. |  |  | * |  |
| New | Thrombocytopenic purpura | easily bruises and sometimes bleeds from the gums and nose; feels weak and has some difficulty with daily activities. |  | * | * | * |
| New | Trigeminal neuralgia | has episodes of severe pain in the face. |  |  | * |  |
| New | Vaginal discharge | has a vaginal discharge that sometimes causes itch. |  |  | * |  |
| New | Varicose veins | has aches in the legs and swelling of the feet when standing for a long period. |  |  | * |  |
| New | Vertigo and balance disorder (Menière, labyrinthitis) | has short spells of dizziness and loss of balance; between spells the person is worried the spells will occur again. |  | * | * | * |
